# Commentary on De Gannes et al. (2013): “Insights into fungal communities in composts revealed by 454-pyrosequencing: implications for human health and safety”

**DOI:** 10.3389/fmicb.2014.00372

**Published:** 2014-07-22

**Authors:** Aurelio Briones

**Affiliations:** Plant, Soil and Entomological Sciences Department, University of IdahoMoscow, ID, USA

**Keywords:** compost, fungi, pathogens, 454-pyrosequencing, diversity

## Next-generation compost fungal ecology

Composting reduces the volume of and transforms spent organic wastes into a valuable soil amendment. In certain situations, composting can literally save lives: during the aftermath of the 2010 earthquake in Haiti, chaos reigned and it didn't take much for sewage and drinking water streams to intermingle, producing tragic results. In this situation, composting of human wastes helped prevent the wider spread of waterborne diseases such as cholera. When properly undertaken, composting contributes to sanitation and the end product is a humus-rich, value-added material that improves the soil. A compost pile supports complex, staged microbiological processes carried out at temperatures that can range from ambient to extremely thermophilic, with important functions carried out by both prokaryotes and eukaryotes. The complex microbiology of compost is ideal for the application of next-generation DNA sequencing (NGS) technology, which offers the needed depth to investigate relationships between composting stages, changes in diversity and succession of abundant and rare microbial populations.

The recent article by De Gannes et al. (2013a) provides one of the first in-depth studies into the microbial ecology of compost fungal communities using NGS and demonstrates its usefulness in addressing basic ecological questions, such as those relating diversity to resource limitation through time under dynamic environmental conditions. A sister publication by the same authors (De Gannes et al., 2013b) showed that prokaryotic diversity in different composts (from wastes derived from bagasse, coffee, and rice) increases as resources become limited, i.e., consumed by microbes. This is generally in line with classical ecological theory, which predicts that diversity is directly proportional to the number of resources at limiting levels within a system (Tilman, 1982). The relationship holds true for prokaryotes through progressive disturbances. i.e., early stage acidification from fermentation of labile organics; intermediate stage heat from intense microbial activity; and late stage production of antibiotics often associated with actinomycetes. Compared to prokaryotes, the diversity-resource relationship of certain fungal communities in the same composts were more constrained by prolonged compost temperatures exceeding 60°C, which is a known limitation of fungal growth (Tansey and Brock, 1978). Similar NGS-based surveys targeting microbial predators and parasites will provide a more complete view of the entire compost trophic structure. Studies such as these across different composting recipes and management schemes will contribute to the synthesis of microbial knowledge grounded on ecological principles. However, these studies also raise questions that NGS-based rRNA gene surveys alone cannot answer, i.e., questions regarding the relationship between community structure and function. Composting is essentially a process of biodegradation and humification. Since mature compost that has undergone sufficient heating is often considered safe to handle, how do humic substances affect microbial functions in general, and virulence of potential pathogens in particular?

The article highlights the detection of potentially pathogenic fungi in compost. Assuming that different types of potential fungal pathogens are present in compost, how does their presence as detected by NGS relate to viability? Composting is a good source of bioaerosols—airborne particles that contain microbes or parts of microbes including endotoxins and mycotoxins. For those constantly exposed to compost dust, there is a significantly higher risk for respiratory, gastrointestinal, and skin problems (Hambach et al., 2012). Therefore, it makes sense to confirm the viability of potential pathogens and to determine how consistently they are detected in composts of different types. Despite the sequencing power of NGS, 454 sequencing only provides presence/absence data and combining 454 with other approaches such as culture-based methods or extracting the active RNA component within the system will give us a better indication of viable populations. On the other hand, metagenomics is well-suited to characterize the core compost microbiome, assuming a certain compost type is associated with a certain set of core populations.

Potentially pathogenic fungi in compost may be a cause of concern. Among the potential pathogens detected in this study, compost-derived *Aspergillus fumigatus* has been demonstrated to cause aspergillosis in susceptible individuals. *A. fumigatus* is an example of a successful fungus in the highly competitive compost environment. Adaptations to saprophytic life in the compost heap may have contributed to its success as a parasite in humans (Rhodes, 2006; Cooney and Klein, 2008). But the medical literature also suggests that aspergillosis is overwhelmingly acquired in hospital settings in immunocompromised patients. Although *A. fumigatus* may adapt to the conditions in mammalian hosts, in most cases it does not possess the machinery to overcome a healthy immune response. Based on the paucity of cases relating compost to other fungal diseases in healthy individuals, this may be true of most the potential pathogens detected in this study.

There are few microbial-based processes that can be undertaken from household to community scales using simple to high technology equipment. Like any process dependent on diverse microbial communities, there is an element of risk that needs to be considered. We cannot totally eliminate potential pathogens from these communities. Individuals with compromised immune systems or who are exposed to the composting environment for prolonged periods need to exercise caution. However, composting is an overall benefit to society—including a good educational and research system to study basic ecological questions that can now be better addressed with NGS as well as an expanded array of “-omics” tools.

### Conflict of interest statement

The author declares that the research was conducted in the absence of any commercial or financial relationships that could be construed as a potential conflict of interest.
